# P27 Carbapenem-resistant *Acinetobacter baumannii*: a challenge in the intensive care units

**DOI:** 10.1093/jacamr/dlaf230.034

**Published:** 2025-12-04

**Authors:** Greta Lila, Rrezarta Bajrami, Yllka Begolli, Aida Duriqi

**Affiliations:** University Clinical Center of Kosova, Prishtina, Kosova; National Institute of Public Health of Kosova, Prishtina, Kosova; University Clinical Center of Kosova, Prishtina, Kosova; University Clinical Center of Kosova, Prishtina, Kosova

## Abstract

**Background:**

Carbapenem-resistant *Acinetobacter baumannii* (CRAB) has become one of the leading causes of healthcare-associated infections globally, particularly in ICUs. Epidemiological studies have reported an alarming rise in CRAB infections, leading to a significant mortality rate ranging from 27.8%–35% in various regions globally.

**Objectives:**

To investigate the incidence of *A. baumannii* isolates in clinical samples of hospitalized patients within University Clinical Center of Kosovo (UCCK), their antimicrobial resistance and detection of resistance genes which circulate in ICUs, as there are no available published data. UCCK serves as the only referral tertiary care centre, with 2500 beds, with approximately 64 000 admissions per year.

**Methods:**

A laboratory-based evaluation study was conducted during 2024 for all samples received from ICUs and other wards in which *A. baumannii* was isolated from infected/colonized patients. Isolation, identification and antimicrobial susceptibility testing followed the standard and automated methods as VITEK^®^ and BD Phoenix ™. Susceptibility for colistin were conducted by manual broth microdilution and interpreted according to EUCAST standards. The presence of genes coding for antibiotic resistance was determined using eazyplex and Unyvero.

**Results:**

From January to the end of December 838 isolates of *A. baumannii* were collected. The highest frequency was from Adult Intensive Care Unit 462(55.13%), Neonatal Intensive Care Unit 87(10.38%), Pediatric Intensive Care Unit 70(8.35%), Infective Disease Intensive Care Unit 48(5.72%), Cardiac Surgery Intensive Care Unit 30(3.57%), Post Intensive Care Unit 20(2.38) and Neurology Intensive Care Unit 14(1.67). Other samples in which Acinetobacter was isolated came from Surgical Block 73(8.71%) with the greatest presence from Plastic Surgery Unit 36(49.3%) and Thoracic Surgery Unit 15(20.54%). Most of the isolates were from respiratory source 456(54.41%), wound swabs 104(12.41), blood culture 70(8.53%) and urine samples 23(2.74%). The lowest number of isolates was found in cerebrospinal fluid 5(0.58%). Polymicrobial isolates were found in 252(30.07%) and monomicrobial isolates in 586(69.92%) of the samples. A significant majority A baumannii isolates 810(96.65%) exhibited resistance to carbapenems and were MDR. Resistance rate to tobramycin was 81.02%, amikacin 90.69% and trimethoprim/sulfamethoxazole 79.23%. This study showed a distribution of NDM, OXA-23, OXA-48 and OXA 24-40 in *A. baumannii* strains. OXA 24-40 is detected most often. Colistin showed sensitivity in 97.85% of isolates.

**Conclusions:**

In this study we report a high CRAB isolates among ICU patients. Colistin remains crucial for treating MDR *A. baumannii* until novel therapeutic options become available in our institution such as eravacycline or sulbactam-durlobactam. Our findings underpin the need for strict adherence to and evaluation of infection prevention and control, and continuous surveillance of CRAB in ICU, especially among the risk groups, in the current study setting and beyond. Further surveillance and research on the evolutionary and epidemiological features of clinical CRAB strains are necessary.

